# A new species of *Besleria* (Gesneriaceae) from the Serranía El Pinche (Cauca), southwestern Colombia

**DOI:** 10.3897/phytokeys.162.55891

**Published:** 2020-10-07

**Authors:** Jhon A. Sánchez-Taborda, Alejandro Zuluaga, Laura Clavijo

**Affiliations:** 1 Fundación Ecohabitats, Popayán, Colombia; 2 Departamento de Biología, Facultad de Ciencias, Universidad del Valle, Cali, Colombia; 3 Grupo de Investigación Ecología y Diversidad Vegetal, Universidad del Valle, Cali, Colombia; 4 Instituto de Ciencias Naturales, Universidad Nacional de Colombia, Apartado 7495, Bogotá, Colombia

**Keywords:** Andes, Cauca, Choco biogeographic, Protective Forest Reserve, San Juan de Micay, taxonomy

## Abstract

A new species of the genus *Besleria* (Gesneriaceae), endemic to the department of Cauca, Colombia, is described and illustrated here. The new species, *Besleria santaclarensis* Clavijo & Sánchez-Taborda, was discovered in the Regional Protective Forest Reserve “Serranía El Pinche”, Cordillera Occidental of the Colombian Andes. *B. santaclarensis* is distinguished by the epedunculate inflorescences, usually in the leafless axils near the base, with up to eight orange flowers, and by the magenta calyx that covers 2/3 of the corolla.

## Abstract (nl)

Una nueva especie del género *Besleria* (Gesneriaceae), endémica del departamento del Cauca, Colombia, se describe e ilustra aquí. La nueva especie, *Besleria santaclarensis* Clavijo & Sánchez-Taborda, se descubrió en la Reserva Forestal Protectora Regional “Serranía El Pinche”, Cordillera Occidental de los Andes colombianos. *B. santaclarensis* se distingue por las inflorescencias epedunculadas, usualmente en las axilas de nudos sin hojas ubicados hacia la base, con hasta ocho flores anaranjadas por inflorescencia y cáliz magenta que cubre 2/3 de la corola.

## Introduction

*Besleria* Plum. ex L. includes 160 species (Clark et al. 2020) of terrestrial herbs, shrubs and small trees that grow in the rainforest understory. It is strongly supported as monophyletic in the tribe Beslerieae (Smith 2000; Roalson and Clark 2006; Clark et al. 2010). Biogeographic analyses of the family suggest that it diversified in the Andes 15 Mya (Perret et al. 2013; Roalson and Roberts 2016). *Besleria* is one of the largest and least known genera among the New World Gesneriaceae; it occurs in most Neotropical rainforests with the highest diversity in the tropical Andes (>100 species), followed by Central America (20 species) (Skog and Boggan 2007; Ferreira et al. 2016). Colombia is the country with the highest diversity of *Besleria*, with more than 80 species (Cortés 2013), followed by Peru with 35 (Kvist et al. 2005), Ecuador with about 20 (Skog and Kvist 1997) and Panama with 15 (Skog 1978). In Colombia, most of the species are found in the humid forests of the Andes and the Choco Biogeographic region, whereas few species grow in the Amazon (Cortés et al. 2017). In the Andes, the largest number of species is found in the Cordillera Occidental of Colombia. For example, *Besleria* is the third largest Gesneriaceae genus with 10 species out of the 96 recorded in northern Valle del Cauca (Clavijo et al. 2014). Likewise, Pedraza and Betancur (2015) recorded five species of *Besleria* out of the 70 Gesneriaceae species collected in the National Natural Park Orquídeas (Antioquia), north of the Cordillera Occidental.

The Serranía El Pinche in Argelia (Cauca) is located in the southern portion of the Cordillera Occidental of the Colombian Andes, and is part of the Munchique-Pinche corridor, a region known for high levels of biodiversity (Paz-B et al. 2018). Biogeographically, the Serranía El Pinche is more similar to the Nudo de los Pastos and the Colombian Massif than to the northern portions of the Cordillera, mainly due to the lower elevation Paramos on the Pacific slopes (Becking 1995). For the first settlers of the Serranía, the conservation of native ecosystems and sustainable rural development were paramount in this biodiverse region. Early settlers inspired in their children the importance of preserving their natural resources by creating alliances that would allow the proper management and establishment of a formal protected area. As a result of this initiative, the Regional Protective Forest Reserve “Serranía El Pinche” was created in 2008. The Reserve is part of the San Juan de Micay river basin that ranges in elevation from 1040 to 3744 meters above sea level; it has 7,256 ha, however, after its expansion it will have in total 11,930 ha. 90.4% of the reserve corresponds to primary forests and 9.6% to perturbed forests, crops and pastures. The Reserve “Serranía El Pinche” and the buffer zone include 913 households (Paz-B et al. 2018).

The upper San Juan de Micay river basin is a unique biodiverse area in the Pacific slopes of the Andes that still has large extensions of primary tropical rainforests (Becking 1995), despite high deforestation rates caused by agriculture and the presence of illicit crops. The preservation of protected areas led by local communities in regions suffering rapid deforestation is essential to conserve species and entire ecosystems. In fact, private and community-owned protected areas have been successful in preserving natural ecosystems in the northern Andes, where biological diversity is high (Joppa et al. 2008; Armenteras et al. 2009; Rodríguez et al. 2013). Therefore, following the legacy of the first naturalists and ecologists that explored La Serranía El Pinche, we aim to contribute to the floristic knowledge of this underexplored rainforest in southwestern Colombia. The plant inventory and discovery of new taxa in this region will provide important information to support the future expansion of the Reserve toward the coast, in the municipalities of Guapi and Timbiquí, achieving a broad elevation coverage from the Pacific coast to the paramos of the Andean highlands.

## Methods

During a rapid ecological evaluation carried out in September of 2017 to characterize the vegetation and establish the baseline for expanding the Regional Protective Forest Reserve “Serranía El Pinche”, we discovered a new species of the genus *Besleria* which is described and illustrated here.

The collections of the new species were processed at the herbarium of the Universidad del Cauca (**CAUP**) and were deposited at the Colombian National Herbarium (**COL**), the herbarium of the Universidad del Valle (**CUVC**) and the Botanical Garden of Medellin (**JAUM**). Specimens of *Besleria* from **COL**, **CUVC**, and **JAUM** were studied to confirm the identity of the species. The photographs were taken with a Nikon camera model D 5300. For the general botanical terminology we followed Beentje (2010) and Moreno (1984).

## Taxonomic treatment

### Besleria santaclarensis

Taxon classificationPlantaeLamialesGesneriaceae

Clavijo & Sánchez-Taborda sp. nov.

A49C9D36-016E-5120-8A00-E7D0744C578B

urn:lsid:ipni.org:names:77211931-1

Figs 1 , 2

#### Type.

Colombia, Cauca: Municipio Argelia, corregimiento Santa Clara, vereda Santa Clara, Reserva Forestal Protectora Regional “Serranía El Pinche”, flanco oriental de la Serranía que se desprende de la vertiente pacífica de la Cordillera Occidental, camino por el borde de la quebrada La Planada, 2°23.938'N, 77°18.863'W, 1620 m. 25 Sep 2017 (fl), *Jhon Alexander Sánchez-Taborda, Álex Cortés, Andrea Borrero, Fernando Joaqui, Andrés Pérez, Erminson Buitrago, Julian Uetochambo* 2552 (holotype: COL!; isotype: CUVC!).

**Figure 1.**
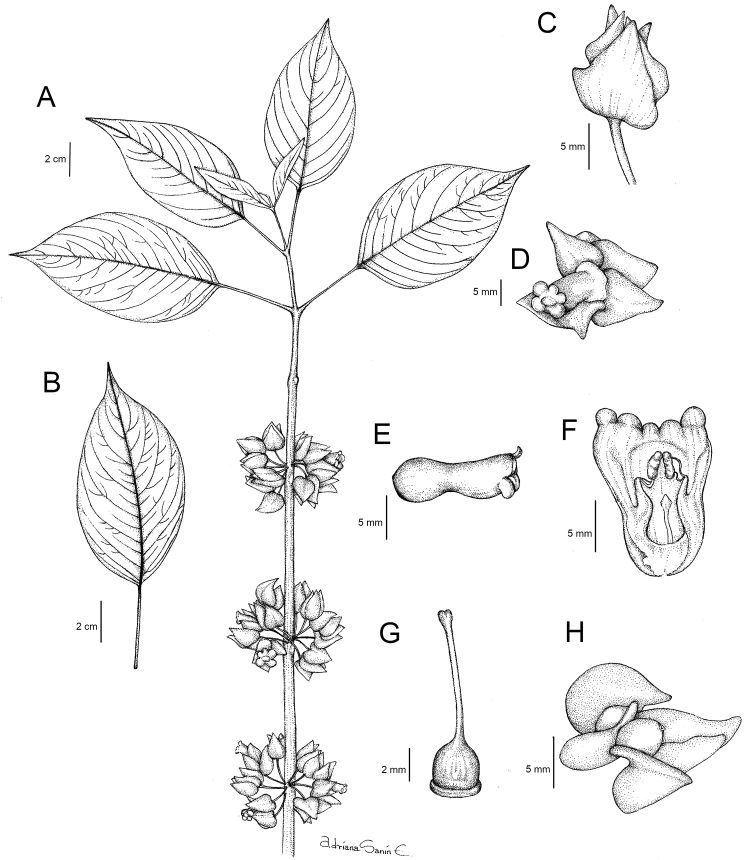
Drawing of *Besleria santaclarensis* Clavijo & Sánchez-Taborda, sp. nov. **A** habit **B** leaf detail, adaxial view **C** calix, lateral view **D** flower, frontal view **E** corolla, lateral view **F** interior view of corolla showing stamens and staminode **G** gynoecium with anular nectary gland **H** fruit, lateral view. Illustration by Adriana Sanín, based on the holotype *Sánchez-Taborda et al. 2552*.

*Besleria santaclarensis* is distinguished by epedunculate inflorescences, usually in the leafless axils near the base of the stem, with up to 8 glabrous orange flowers, and magenta glabrous calyx that covers 2/3 of the corolla.

**Figure 2.**
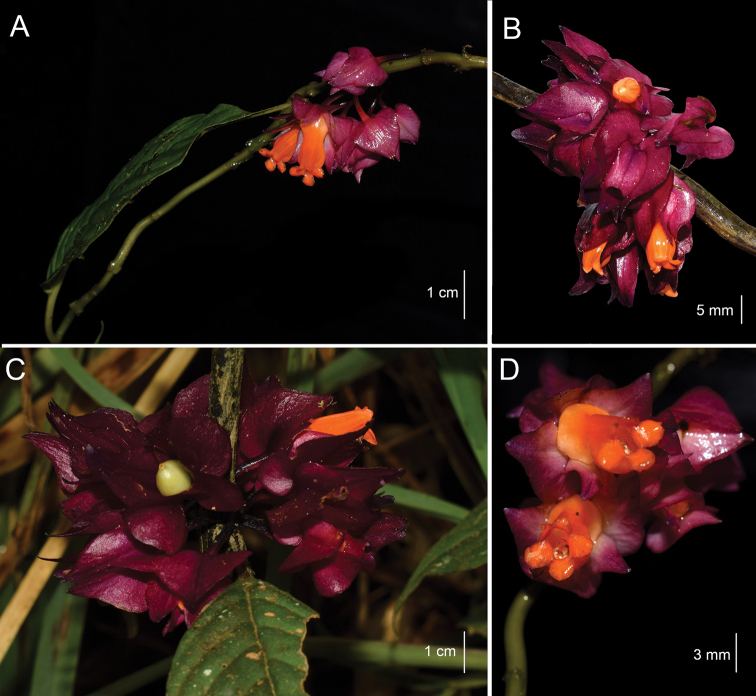
*Besleria santaclarensis* Clavijo & Sánchez-Taborda, sp. nov. **A** branch showing leaf and inflorescence **B** inflorescence showing flower in lateral view **C** inflorescence showing an immature fruit **D** frontal view of the corolla. (Photographs by Jhon A. Sánchez-Taborda from the paratype).

Terrestrial subshrub, 1–1.5 m tall. Stem erect, sometimes scandent, branched, terete in cross section, 1.9–4 mm diam., subwoody, green, surface smooth to longitudinally striated, strigose toward the apex, trichomes < 1 mm long, unbranched, white; internodes 1.7–5.8 cm long. Leaves opposite, equal in a pair, sometimes subequal, older leaves usually caducous; petiole 1.6–4.9 cm long, slightly winged in cross-section, glabrate in basal leaves, strigose in apical leaves, trichomes < 1 mm long, white; blades elliptic, coriaceous, papyraceous when dry, 6.4–17.6 × 2.8–6.8 cm, green and glossy adaxially, olive green abaxially, apex acuminate, base cuneate, sometimes oblique, margin entire, glabrate on both surfaces, 7–10 pairs of secondary veins, obscure adaxially and raised abaxially with scarce and whitish indument, higher order of venation only evident abaxially. Inflorescence a pair-flowered cyme, axillar, usually in the leafless axils near the base of the stem, up to 8 flowers per inflorescence; peduncle and bracts absent. Pedicel oblique to perpendicular relative to the stem, 8.6–17.1 mm long, maroon, glabrous. Calyx magenta, membranous, persistent in fruit, venation evident, reticulated; calyx lobes 5, 4 nearly equal, free, apex acute, base truncate to cordate, margin entire, glabrous on both surfaces, ventral and lateral lobes 9–13 × 6.2–10.6 mm, ovate, dorsal lobe 10.1–11.9 × 4.3–5.3 mm, oblong; Corolla zygomorphic, protandrous, slightly gibbous, thick, 14.4–15.4 mm long, orange, glabrous; corolla tube slightly constricted above the base and then slightly ventricose ventrally, oblique relative to calyx, 12.3–13.5 mm long, 5.7–6.2 mm at its widest part, constriction above the base 3.7–4 mm diam., base 5.4–6.8 mm wide; throat 2.3–2.8 mm diam., inner surface with glandular trichomes; corolla lobes 5, subequal, orange, spreading, ovate, apex rounded, margin entire, glabrous on both surfaces, ventral lobe 1.4–2.6 × 2.4–3.1 mm, lateral lobes 2.5–3.4 × 2.4–3.9 mm, dorsal lobes 0.8–1.1 × 1.8–2.3 mm. Androecium of 4 stamens, didynamous, included; filaments 7.1–10.1 mm long, adnate to the corolla tube for 1.9–2.6 mm, forming a sheath, glabrous, coiling after anthesis, staminode 4.9–5.2 mm long; anthers reniform, 1.2–1.4 × 1.1–1.7 mm, coherent by the apex and lateral walls, dehiscence by longitudinal slits. Gynoecium with an annular nectary gland, 0.5–0.7 mm tall, glabrous; ovary superior, 3–3.1 × 2.5–2.7 mm wide, rounded, glabrous; style included, 6.4–6.8 mm long, glabrous; stigma bilobed. Fruit a berry olive green; seeds numerous.

[Measurements from flowers during the mature gynoecium phase.]

#### Distribution and ecology.

**Figure 3.**
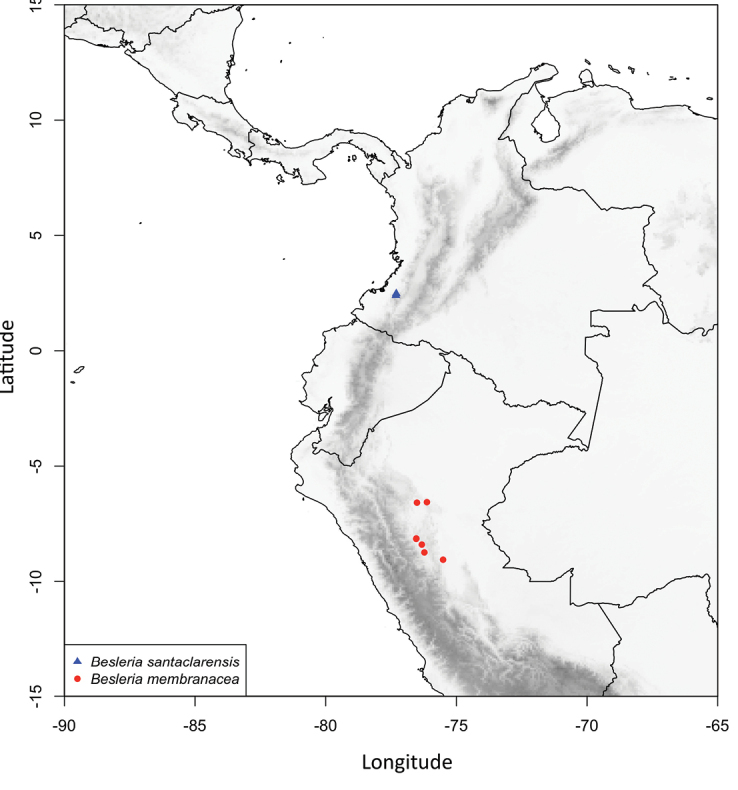
Distribution map of *Besleria santaclarensis* and *Besleria membranacea*.

*Besleria santaclarensis* is endemic to Colombia and known only from the type locality in the municipality of Argelia (Cauca) in the Regional Protective Forest Reserve “Serranía El Pinche” and surrounding areas (Fig. 3). This species grows in the lower montane rainforest (Holdridge 1967) between 1300 and 1600 m above sea level, on the Pacific slope of the Cordillera Occidental of the Andes. It is frequent in open areas and in forest clearings, usually near to crops, pastures and remnants of secondary and riparian forests, whose canopies reach up to 35 m and 25 m, respectively. These forests have typical Andean floristic elements, represented by the genera *Saurauia* Willd (Actinidaceae), *Schefflera* J.R. Forst. and G. Forst. (Araliaceae), *Axinaea* Ruiz and Pav. (Melastomataceae), *Ladenbergia* Klotzsch (Rubiaceae), *Wettinia* Poepp, *Socratea* H. Karst and *Iriartea* Ruiz and Pav. (Arecaceae), *Cyathea* Sm and *Alsophila* R. Br. (Cyatheaceae).

#### Phenology.

*Besleria santaclarensis* has been found in flower in February and September and in fruit in February.

#### Etymology.

*Besleria santaclarensis* is named after the type locality, the Santa Clara village, in the municipality of Argelia, Cauca. The territory currently known as Argelia and areas surrounding El Pinche mountain range were initially inhabited by the *Guapios* indigenous people, until the arrival of the colonists who named it the Agua Clara path, due to the streams of crystalline waters present in the area. Afterwards, during evangelization, a Franciscan priest changed the name to Santa Clara because of the fertility of the land, which he called holy (Alveiro Bolaños, personal communication).

#### Preliminary conservation status.

*Besleria santaclarensis* is only known from the Regional Protective Forest Reserve “Serranía El Pinche” and surrounding areas. Although the Reserve is a protected area, deforestation and soil degradation caused by agriculture and livestock systems, as well as the presence of illicit crops in the surrounding areas, may have a significant impact on the integrity of this narrow endemic species. *B. santaclarensis* is present in mature forests, but it is also frequent in open areas in remnants of secondary and riparian forests, suggesting it is resilient and capable of thriving in disturbed areas. A preliminary designation of Endangered (EN) category is provided, according to the criterium B2ab (IUCN 2012, 2017), based on the small population of the species, with an estimated area of occupancy of less than 20 km^2^, and the continued habitat loss, due to the high concentration of illicit crops in the area (UNODC 2019).

## Discussion

*Besleria santaclarensis* can be readily distinguished from its congeners by the epedunculate inflorescences, usually in the leafless axils near the base of the stem, with up to 8 glabrous orange flowers, and magenta glabrous calyx that covers 2/3 of the corolla. *B. santaclarensis* is similar to *B. membranacea* C.V. Morton in their foliage, their flowers with membranous and glabrous calyx, and their slightly gibbous and glabrous corolla tube. *B. membranacea* is endemic to Peru, particularly to the Amazon and the eastern foothills of the Andes, in the Departments of Loreto and San Martín, mainly in the basin of the Huallaga and Aguaytía rivers, between 260 and 880 m of elevation (Salinas and León 2006). *B. santaclarensis* differs from *B. membranacea* by shorter petioles (1.6–4.9 cm vs. (3.2–)6.5–11.6 cm), inflorescences with up to 8 flowers (vs. up to 4), shorter pedicels (8.6–17.1 mm vs. 13.1–20 mm), calyx base truncate to cordate (vs. never cordate), larger calyx lobes (9.0–13.0 × 4.3–10.6 mm vs. 5.8–9.3 × 1.6–4.7 mm) that cover up to 2/3 of the corolla (vs. covering up to 1/2 of the corolla), and corolla tubes shorter (14.4–15.4 mm vs. 14.9–18 mm long) and orange (vs. yellow and white). Additionally, *B. santaclarensis* is similar to an undescribed species collected in the Province of Zamora-Chinchipe, Ecuador, by Dr. John Clark (*Clark et al. 10815*). The two species share the inflorescences with several orange flowers with magenta ovate calyx lobes, but differ in that *B. santaclarensis* has glabrous calyx (vs. pilose) and slightly gibbous and glabrous corolla tube (vs. gibbous and pilose).

With the discovery and description of *B. santaclarensis* we aim to contribute to the floristic knowledge of this underexplored rainforest in southwestern Colombia, and to provide new information to support the future expansion of the Reserve that will warrant the conservation of this and many other species.

**Additional specimens examined (paratypes).** Colombia. Cauca: municipio Argelia, corregimiento Santa Clara, vereda El Pinche, zona aledaña a la Reserva Forestal Protectora Regional “Serranía El Pinche”, flanco oriental del Cerro Pinche, camino entre el Plateado y Guapi, en zona cercana a cultivos y pasturas con algunos relictos de bosques secundarios y riparios de la quebrada El Pinche, 2°28.809'N, 77°18.014'W, 1475 m. 11 Feb 2018 (fl, fr). *Jhon Alexander Sánchez-Taborda, Fernando Joaquí and Andrés Pérez* 2938 (JAUM).

## Supplementary Material

XML Treatment for Besleria santaclarensis
